# 

**Published:** 1889-04-06

**Authors:** 


### The Hospital

530

A Weekly Institutional Journal of

### Science, Medicine, Nursing, and Philanthropy.

EDITED BY

HENRY C. BURDETT,

AUTHOR OF "HOSPITALS AND THE STATE"; "PAY HOSPITALS OF THE WORLD"; "COTTAGE HOSPITALS, GENERAL, FEVER, AND CONVALESCENT, WITH FIFTY BEDS AND UNDER: THEIR ORGANISATION, MANAGEMENT, AND WORK,' "HELPS TO HEALTH"; ETC.

ACTING EDITOR.

GEORGE W. POTTER, M.D.,

AUTHOR OF PAPERS ON "BACTERIOLOGY"; SCARLET-FEVER"; "RHEUMATISM"; ETC., TC.

### INDEX TO VOL. VI.

(April 6th, 1889, to September 28th, 1889).

**FOR 1889.**

SURGEON GEN'L'S OFFICE LIBRARY 13067

LONDON:

PUBLISHED BY "THE HOSPITAL" (Limited), 139 and 140, SALISBURY COURT, FLEET STREET, E.C.

W1 H069X

### INDEX TO VOL. VI. 1889.

Abstainers and Life Assurance, 15, 47
After-Care Association, 262
All-year Weekly Collection of Liverpool Hospital Saturday Fund, 231
Almsgiving, Indiscriminate, 404
Ambulance Classes at the People's Palace, 343
Ambulance for Injured Horses, 217
Ambulance Pupils at Hastings, 230
Ambulance, Street Service for London, 12, 73, 119
——— Mr. Ryan's Scheme, 73
Amines Process for Purification of Sewage, 271
Anæsthetics and Antiseptics, 135
Anæsthetics, Moral Effects of, 89
Anatomy, a Textbook of Pathological,
Annual, Burdett's Hospital, 80, 134
Anthrax and Vaccination, 358
Anthropological Congress, 358
Antiseptic Dressings, 301
Anti-Vaccinators and the Royal Commission, 202
Arsenic in Glycerine, 390
Asylum Reports, 132, 229, 258, 277, 293, 309, 338, 354
Atkinson, A. G. B., on Student Life in Edinburgh, 90
Authority, Medical, 89
Awards of Metropolitan Hospital Sunday Fund, 285

Bakewell, Dr., on Leprosy and Vaccination, 295
Baltimore, Johns Hopkins Hospital at, 92, 127, 230, 314, 330, 346, 362
Barmaids and the Young Women's Christian Association, 249
Barnett, Rev. and Mrs. S. A., on Practical Socialism, 28
Barnsley Beckett Hospital, Demonstrations in Aid of, 342
Barrow Workmen and the Hospital Concert, 342
Bath Hospital and Convalescent Home, 263
Bath Hospital Saturday Fund Instituted, 178
Battle, the Law of, 361
Beef-tea and How to Make it, 355
Belfast Royal Hospital, 359
Belvidere Infectious Hospital, 70
Berlin, Shelter for Destitute Poor in, 359
Biddenden Maids, the, 376
Billings, Dr. J. S., on the Johns Hopkins Hospital, 314, 330, 346, 362
Birmingham Hospital Saturday Fund, 278
Bishop of London's Fund, 117
Blackheath Hill Boys' Orphanage, 199
Boarding Out of Pauper Children, the, 340, 356
Books, the Burden of, 11
Brabazon Home of Comfort, 135
Bradford Children's Hospital, 86
Brain and Nerve, Curiosities of, 341
Bread and Food Reform League, 278
British Dental Association, 342
British Medical Association, Mr. Wheelhouse's Address to the, 319
——— and the *British Medical Journal*, the, 329
——— and Disinfectants, 375
British Nurses' Association, 209, 249
——— What will Trained Nurses gain by Joining the, 237
Bristow, Dr., on The Hospitals Association, 154
Brompton Hospital, Princess Christian at, 263
Bronchial Tubes, Foreign Bodies in, the, 308
Brown-Sequard, Dr., and Elixirs of Life, 297
Browne, Sir James Crichton, on the Pleasures of Imagination, 354
Burdett, Mr. Henry C., on the Relief of Distress, 299
Burdett's Hospital Annual, 80, 134
Buried Alive in Bohemia, 327

Cabdrivers' Benevolent Association, 121
Calcutta and Sanitation, 310
Campbell, Dr. Harry, on the Causation of Disease, 68, 114, 148
Cancer Cure, 101
——— Diagnosis of, 308
Carlsbad, 105
Catalepsy or Death, 111
Causation of Disease, the, 68, 114, 148
Cemeteries of London, Returns respecting the, 310
——— Overcrowded, 386
Central London Throat and Ear Hospital, 247, 278
Charing Cross Hospital Medical School, Session 1889-90, 378
Charity and the Prince and Princess of Wales, 53
Charity Organisation Society, Memorandum of, 71
Charity Organisation Society, Petition of, 63, 273, 278
Chemical Formulæ, 210
Child-Life, a Magisterial Valuation of, 105
Children and Creeds, 335
Children, Great Ormond Street Hospital for, 115
Children, How they are Poisoned, 201
Children, How to Give Medicine to, 7
Children's Country Holidays Fund, 196, 215, 290, 308, 313, 335, 356
Children's Protection Bill, 361
Children, the Society for the Prevention of Cruelty to, 11, 361.
Chloroform and the Heart, 8, 247
Cholera, 23
Cholera, Summer Diarrhœa and, 221
——— on Some Points connected with:
Its Definition and Symptoms, 305
Theories Examined, 321
A Modern Theory, 337
Common Symptoms, 357
——— the Cause of, 141
Civilisation and the natives of Tonga Islands, 310
Clapham, Women Physicians at, 403
Clark, Sir Andrew, on Hospitals, 177, 191
Clergy, the, and Hospital Sunday, 159
Clerks, Combining, 281
Clifford, E. T., on Abstainers and Life Assurance, 47
Cobbe, Miss F. P., on Hospitals, 342
Consolation, Words of, 272, 288, 304
Contagious Diseases Act in India, Result of Suspension of the, 326
Convalescent Children, Miss Derham's Home for Semi-, 358
Convalescent Home for Friendly Societies, 294
——— Reports, 295, 311
Convalescent Home for Ladies, 118
Convalescent Home at Moretonhampstead, 198
Convalescent Home for Working Men at St. Margarets, 279
Convalescent Hospitals, 24
Countess of Dufferin's Fund, the, 81
Country Holidays for Town Children, 196, 215, 290, 308, 313, 335, 356
Coventry Hospital and Working Men Governors, 198
Coventry Provident Dispensary, 247
Cow's Milk, a Poison in, 201
——— and Consumption, 202
Creighton, Dr., a Strange Chapter of Medical History by, 213
Crime and Criminals, 289
Critics of the Hospital System, 160

Daisy and Buttercup Fund, 269
Dale, Dr. R., an Epitome of Surgery by, 34
Damien, Father, 109, 185, 198
Damien Memorial, the, 185
Deaf and the Dumb, the, 405
Deaf, What should they do? 318, 329
Dean's Opinion, a, 369
Death by Electricity, 150, 285
Death Rate at Manchester, 102
Dental Association, British, 342
Dental Department at Guy's, 214
Derham's Home for Semi-Convalescent Children, Miss, 358
Diarrhœa and Diphtheria, Report on, 70
——— Bacterial, at Leicester, 292
——— Summer, and Cholera, 221
Dinners for Children at St. Gabriel's, Canning Town, 246
Diphtheria in Northamptonshire, 358
Disease and Will-Making, 361
Disease, the Causation of, 68, 114, 148
Disinfectants, 345
Disinfection, Successful, 64
Dispensary at St. Bartholomew's Hospital, 215
Distress, How to Provide for the Winter's, 299
Doctors and Paupers, 345
Doctors and the Compulsory Notification of Infectious Diseases, 287, 295
Doctor's Pulpit, The:
Man and His Enemies—
——— The Pinafore Period, 3
——— Breaking to Harness, 19
——— A Youth's Catechism of Health, 35
——— Barriers against Marriage, 65
——— The Wager of Battle, 83
——— Invisible Companions, 99
——— A Faint Heart, 113
——— Home Difficulties, 131
——— Festina Lente, 147
In Nature's School, 179
——— Object Lessons, 195
——— Room to Breathe, 211
——— Wild Roses, 227
——— First the Blade, 241
——— Dewdrops, 257
——— Beauty and Use, 275
——— Thistledown, 291
——— Trained or Strained, 307
——— Ex Nihilo, 323
——— "Dodging" the Weather, 339
——— Closed Records, 353
The Medical Mind and Conscience, 371
Quiet Habits: Long Life, 385
Fun at Fifty, 401
Doctors, Two Famous, 255
Donegal Fund, the, 98
Douglas Lithgow, R. A., LL.D., on Heredity, 125
Dressings, Antiseptic, 301
Drysdale, Dr., on the Evils of Large Families, 327
Dublin Hospital Board, 343
Dublin Hospitals, Report on, 278
Dufferin Maternity Hospital, Burmah, 343
Dundee Royal Infirmary, 182
Durham Samaritan Society, 327
Dyspeptics, Pure Air for, 233

Economy in Hospitals, 25, 278
Edinburgh Castle, Good Friday Concert at, 77
Eggs, Fresh, 409
Education by Picnic, 389
Electricity, Death by, 150, 295
Electric Tramcars, 96
Enlargements of Hospitals, 102, 119, 183, 327, 342, 343
Epilepsy and Paralysis, Hospital for, 183
Epilepsy, Procursive, 180
Ether Drinking in North of Ireland, 393
Examinations in Elementary Schools, 1
Execution by Electricity, 150
Expenditure of Hospitals, 97, 278
Eye and Ear Infirmary, Dublin, 106
Eye Hospital at Oxford, 263
Eye, the, Sir J. Paget on, 263

Fever Hospitals, the Nursing in, 317
Firth, Mr. J. F. B., Death of, 377
Fish, Torturing and Starving, 325
Flitwick Water, 221
Food in Summer, the Dangers of, 153
Football, a Plea for, 121
For the Right, 112
Foundling Hospitals in Russia, 135
France and the Population Question, 359
French-Sheldon, M., "Herbert Severance," by, 74
Friendly Societies' Convalescent Home, Proposal for, 294
Froude, J. A., "The Two Chiefs of Dunboy," 253

Gairdner, Dr., Addresses and Memoirs by, 197
Gambling, Convocation and, 233
General Practitioner and Hospital Abuse, the, 313
General Practitioner, the Revolt of the, 281
Genius, Characteristics of, 74
Gibson, Dr., the "Characteristics of Genius," by, 74
Gilbart-Smith, Dr., on Should Out-Patients Pay, 98
Gladstone, Mr., and the Prince of Wales, 85
Gladstone's, Mrs., Home in Epping Forest, 225
Glasgow Charities, Bequests to, 391
Gould, Dr. Pearce, and Medical Students, 369
Graphophone, 9
Great Ormond-street Hospital, 115
Grosvenor Hospital, 71
Guy's Hospital, Dental Department, At, 214
Guy's Hospital, In the Surgery at, 138
Guy's Hospital Medical School, Session 1889-90, 378

Halifax Borough Fever Hospital, 294
Halifax Infirmary, Report on Overcrowding of, 198
Hanwell Schools and Ophthalmia, 137, 214
Headaches, Nervous, 43
Headley, Rev. T. G., on a Physiological Sunday, 240
——— on Religious Excess, 269
Health and French Journalism, 100
Health Congress, 87
Hearts, Weak, 43
Heat and Hats, 217
Heathcote Hospital for Infectious Diseases opened, 150
Heredity, 125
Hereford Dispensary, Report of the, 311
Her Heart's Mistake, 13, 29, 45, 75, 91, 107, 123, 139, 156, 188, 203, 219, 235, 251, 267, 283, 298, 315, 331, 348, 363, 381, 394, 410
High Game—Is it Poisonous? 228
Home Hospitals Association, 135
Homœopathic Hospital, London, 87
Hospital Abuse, the General Practitioner and, 313
Hospital Annual, Burdett's, 80, 134
Hospital Board for Dublin constituted, 343
Hospital Economy, 25, 278
Hospital Expenditure, Variations in, 21
Hospital for Women, 93
Hospital Reports, 8, 24, 25, 41, 70, 71, 86, 87, 106, 118, 150, 151, 182, 183, 199, 230, 231, 246, 247, 279, 295, 311, 326, 342, 343, 358, 359.
Hospital Saturday Fund, Liverpool, 231; Birmingham, 278; Bath, 178
Hospital Sunday and the Clergy, 159
Hospital Sunday Fund, the Insufficient Amount of the, 176
——— Sir Andrew Clark pleads for the, 177
——— Awards, 285
Hospital System and its Critics, the, 160
Hospital Workshop, The—
With the Sick Children, Great Ormond-street, 115
In the Surgery at Guy's, 138
A Saturday Night at the London Hospital, 186
The Throat Department at St. Mary's Hospital, 218
The National Hospital for the Paralysed and Epileptic, 234

Hospital Workshop, The—
At the Royal Westminster Ophthalmic Hospital, 244
Out-Patients at St. Thomas's, 282
Out-Patients at the Brompton Hospital for Consumption and Diseases of the Chest, 347
Out-Patients at St. Bartholomew's,
Hospitals, Abuse of, 9
Hospitals Association, the, 8, 12, 40, 98, 143, 154
——— the Street Ambulance Branch, 12, 73, 119
Hospitals and Medical Charities of London, Summary of a Year's Work at, 171
Hospitals and the House of Lords, 63
Hospitals and their Expenditure, 97, 242, 278
Hospitals and Workmen's Contributions, 33, 79 109
Hospitals for the Insane, 393
Hospitals of London, the, 273
Hospitals without Medical Schools, 242
Hotels of England and their Critics, 239, 245
Howard Association, Report of, 333
Hull Infirmary and Working Men, 391
Hydrophobia, 129, 145, 193, 326
Hypnotism, 151, 409
Hysterical Men, 117

Idiots, the Improvement of, 144
Illingworth, Dr. C. R., on How to Give Medicine to Children, 7
Imagination, the Pleasures of, 354
India, National Association for Supplying Female Medical Aid to the Women of, 81
Infantile Assurance, 153
Infectious Disease, Absence of Clinical Instruction in, 310
Infectious Diseases, Compulsory Notification of, 287, 294
Infectious Hospital at Salford, 40
——— at Nottingham, 183
Infectious Hospitals of London, Dr. Seaton on the, 230
Insane, Glances at, in other Lands, 23
Insanity, Progress in the Study of, 343

Jenner and Vaccination, 213
Johns Hopkins Hospital, 92, 127, 230
Johns Hopkins Hospital, the Plans and Purposes of the—
Instructions and Cardinal Principles, 314
Cardinal Principles, 330
A Nurse Training School, 346
The Hospital and Medical Education, 362
Journalism of France and its Health Results, 106

Kidney, a Stone in, 65
King's College Hospital Medical School, Session 1889-90, 378
Klein, Dr., "the Bacteria in Asiatic Cholera," by, 141

Ladies, Convalescent Home for, 118
Lawrence-Hamilton, Mr., on Torturing and Starving Fish, 325
Leeds, Red-tape at, 151
Left-leggedness, 387
Leicester Dispensary, 102
Leisure and Temper, 11
Leper Colony at Robben Island, 374
Leper Hospital in the West Indies, 214
Leprosy and Vaccination, 295
Leprosy in England, 198, 250
Leprosy, Is it Infectious? 181
Life Assurance and Abstainers, 15, 47
Life in Modern Asylums, 44, 67
Liverpool Convalescent Institution, 230
Liverpool Hospital Fund Awards, 326
Llandrindod Hospital, 231
Lockjaw, Cases of, 326
London Hospital and the National Pension Fund for Nurses, the, 306
London Hospital, a Saturday Night at the, 186
London Hospital, Prize Day at, 253, 378
London Hospitals, the Peers on, 273
London Sewage, 249
London, Street Ambulance Service for, 12, 73, 119
Longevity, Dr. Robson Roose on, 182
Luckes, Miss, on "What will Trained Nurses Gain by Joining the British Nurses' Association," 237
Lyon, J. B., on Medical Jurisprudence for India, 243

Macdonald, Dr., on Infantile Mortality and Life Assurance, 153
Mackenzie, Sir Morrell, on the "Cracking" Period of the Voice, 295
Mackinnon Memorial Hospital, 359
Manchester Infirmary Board, Report of, 279
Mandragora, 397
Maybrick Case, the, 303
Meath Hospital, 40
Medical Freshman, the, 367
Medical Jurisprudence for India, 243
——— Mind, the, and Conscience, 371
Medical Service for England, a Public, 207
Medical Schools of London, Arrangements for Session, 1889-90, 378
Medical Staff Corps, Volunteer, 102

Medical Students, Mr. Pearce Gould on, 369
——— Student, the, as the Nurse Sees Him, 370
Medicine, Address and Memoirs Bearing on the History of, 197
Medicine Men of the World, 39, 69
Medicine, the School of, for Women, 208
Method, Originality of, 351
Metropolitan Hospital, 247
Metropolitan Hospital Sunday Fund, the, 176, 285
Middlesex Hospital, 359
——— Medical School, Session 1889-90, 378
Milk, Dangers of Raw, 149
Money, the Want of, 281
Monomania, Dr. Greene on, 343
Montrose, Munificent Bequest to, 406
Moore, Sir William, on Opium and its Votaries, 5
——— on Summer Diarrhœa and Cholera, 221
——— on Nightmare, 324
Morality, Progress by, 43
Muddled Medicals, "Mems" for, 372

Nansen, Dr., his Message, 399
National Home Reading Union, the, 342
National Hospital for the Paralysed and Epileptic, 133
National Pension Fund for Nurses, 122
——— the London Hospital and the, 306
Newcastle Hospital Sunday Fund, 406
New Convalescent Homes, 198, 214
New Hospital for Women, the, 358
New Hospitals, 9, 24, 40, 41, 70, 86, 87, 93, 103, 150, 182, 183, 198, 199, 230, 262, 263, 279, 294, 327, 358
Newsholme, Dr., on Vital Statistics, 146
Nightingale, Florence, 259
Nightmare, 324
North-Eastern Hospital for Children, the, 151
Nottingham and Infectious Hospitals, 183
Nottingham General Hospital, 71, 103
Nun of Kenmare, the, 142
Nurses, Trained, What will they Gain by Joining the British Nurses' Association, 237

Ophthalmia at Hanwell Schools, 137, 214
Opium and its Votaries, 5
Oral Instruction of the Deaf and Dumb, Association for, 247
Originality of Method, 351
Out-Patients and Paupers, 345
Out-Patients at St. Thomas's, 282
——— at St. Bartholomew's, 365
——— at the Brompton Consumption Hospital, 347
Out-Patients, Should they Pay, 98
Overfeeding, 73

Paddington Workhouse, Sale of Pauper's Work at, 194
Paget, Sir James, on the Eye, 263
Palpitation, The *Medical Annual* on the Treatment of, 313
Paralysed and Epileptic, The National Hospital for, 234
Pasteur Institute, the Proposed, 185
Pasteurism, a Protest Against, 256
Pauper Children, the Boarding Out of, 340, 356, 388
Penny-a-Week Collections for St. Helens Hospital, 230
Penny-a-Week Scheme, the, 70, 79
People, The, 51
Petition of Charity Organisation Society to the House of Lords, 63, 273, 278
Physical Development Society, 231
Pick-me-ups, The *St. James's Gazette* on, 211
Pleuro-Pnuemonia, 86
Poetry, 52, 176, 270, 276, 286
Policemen and the St. John's Ambulance Association, 326
Poor in Berlin, Shelter for Destitute, 359
Population Question in France, 359
Poverty and Happiness, 399
Practical Socialism, Rev. and Mrs. S. A. Barnett on, 28
Preachers and Subjects on Hospital Sunday 1889, Some, 189
Prince and Princess of Wales and Charity, 53
Prince, The, 49
Princess, The, 50
Prisons, the Report of the Howard Association on our, 333
Psychical Society, the, 151
Public Servants of the Future, 383

Rawlings, B. B., on the Working Classes and the Hospitals, 109
Readings for the Sick, 272, 288, 304, 320, 336, 352
Registration of Nurses, Argument against the, 209
——— Miss Luckes on the, 237
Religious Intoxication, 73, 224, 269
Rentoul, Dr. R., and Paupers, 345
Reviews: "Guide to Stretcher and Bearer Company Drill, 2, 48
——— "What must I do to get well," 36
——— "Herbert Severance," 74
——— "Characteristics of Genius," 74
——— "Board School Laryngitis," 106

Reviews: "For the Right," 112
——— "The Cause of Cholera," 141
——— "The Nun of Kenmare," 142
——— "Heredity," 125
——— "Hospital Annual," 80
——— "The Son of a Star," 226
——— "The Two Chiefs of Dunboy," 253
——— "Songs of the Spindle," 256
——— "Addresses and Memoirs bearing on the History and Progress of Medicine during the last Hundred Years," 197
——— "A Strange Chapter of Medical History," 213
——— "Crime, the Convict and the Prison," 289
——— "The Elements of Vital Statistics," 146
——— "Daphne's Daring," 338
Richardson, Dr., as a Novelist, 226
Richardson, Mr. John, his Bequests to Hospitals, 198
Robben Island Leper Colony, 374
Roche's Protest against Pasteurism, 256
Roose, Dr. Robson, on Longevity, 182
Royal Masonic Institution for Boys, 262
Royal Maternity Charity, 118
Royal Sea Bathing Infirmary, 135
Royal Westminster Ophthalmic Hospital, 244
Ryan, Mr. Thomas, on National Pension Fund for Nurses, 122
Ryan, Mr. Thomas, on a Street Ambulance Service for London, 12, 73, 119

Salford Convalescent Home, Enlargement of, 134
Salford Infectious Hospital, 40
Salvation Army Hospital, a, 297, 327
Samaritan Free Hospital, 86, 262
Sansom, Dr., on the Registration of Nurses, 209
Schools, Examinations in Elementary, 1
School of Medicine for Women, the, 208
Science and the Royal Society, 95
Seashell Mission, 199
Sea-sickness, 266
Seaton, Dr., on the London Infectious Hospitals, 230
Servants, Metropolitan Association for Befriending Young, 202
Sewage of London, 249, 295
Sewage, the Amines Process for Purification of, 271, 413
Shock, 73
Sibley, Dr., on Left-leggedness, 387
Sick Children's Hospital, Great Ormond-street, 150
Skim Milk as Food, 27
Skin Hospital, London, 230
Sleeplessness, 377
Snakebite Cured, a, 180
Snakebite, Strychnia in, 18
Socialism, Practical, 28
Society for the Prevention of Cruelty to Children, 11
Soldiers' Daughters' School, Hampstead, Prize-Day at the, 205
Squint and Spectacles, 292
St. Bartholomew's Hospital Medical School, Session 1889-90, 379
St. George's Hospital Medical School, Session 1889-90, 379
St. John's Ambulance Association and Policemen, 326
St. John's Hospital for Diseases of the Skin, 128
St. Katherine's Hospital, 311
St. Mary's Hospital, 24, 135, 375
——— Throat Department at, 218
——— Medical School Session, 1889-90, 378
St. Thomas's Hospital Medical School, Session 1889-90, 379
Stanley Hospital, Liverpool, Bazaar in aid of, 182
Statistics, the Elements of Vital, 146
Steak-Pie, a Story of, 228
Street Accidents in London, Mr. Thomas Ryan on the Transport of, to Hospitals, 12, 73, 119
Stretcher and Bearer Company Drill, Guide to, 2, 48
Student Life in Edinburgh, 90
Student, the, as the Nurse sees Him, 370
Student's Column, the, 6, 20, 34, 243
Sudbury, St. Leonard's Hospital, 359
Suicides, Would-be, 265
Summary of a Year's Work in the Hospitals and Medical Charities of London, 171
Summer Diarrhœa and Cholera, 221
Sunday, a Physiological, 223, 240
Surgery, a Complete Compendium of the Science and Art of, 34
Surgical Treatment, Unskilful and Negligent, 247
Sweating, 41

Teachers of Medicine, 351
Thames Church Mission, 204
Thriftlessness and Suicide, 345
Tight-Lacing, New Diseases from, 27
Tonga Islanders and Civilisation, 310
Toughened Nerves, 377
Twelve Hundred Miles under Difficulties, 37
Typhoid, 86
Tyrotoxicon, 201

University College Hospital, 8
——— Medical School, Session 1889-90, 380

1306 TH

Unqualified Assistant, the, 380

Vaccination, 41, 89, 213
—— Commission on, 70, 183, 202, 217
—— and Leprosy, 295
Van Praagh, Mr. William, and Defects of Speech, 247, 278
Victoria Eye and Ear Hospital, Opening of, 358
Vital Statistics, the Elements of, 146
Vivisection, 4, 22, 38, 118, 131, 192
Voice, Sir M. Mackenzie on the "Cracking" Period of the, 295

Waterlow, Sir S. H., and the London Street Ambulance, 73
Waterson, Staff-Sergt. W. K., Guide to Stretcher and Bearer Company Drill, 2 48
Westminster Hospital Medical School, Session 1889-90, 380
Wheelhouse, Mr., his Address to the British Medical Association, 319
Will-Making and Disease, 361
Wines, Mr. F. H., on Crime, the Convict, and the Prison, 289

Within the Wards:
—— Vivisection, 4, 22, 38
—— The Making of New Features, 38
—— A Stone in the Kidney, 66
—— Cancer Cure, 101
—— Scrofulous Glands, 101
—— Lung Cavity opened Surgically, 101
—— Hysterical Men, 117
—— Tumour, 133
—— Dangers of Raw Milk, 149
—— Tendon Grafting, 149
—— Procursive Epilepsy, 180
—— A Snakebite Cured, 180
—— Is High Game Poisonous? 228
—— A Steak-Pie Story, 228
—— Leprosy in England, 250
—— About Chemical Formulæ, 210
—— Bacterial Diarrhœa at Leicester, 292
—— Squint and Spectacles, 292
—— Foreign Bodies in Bronchial Tubes, 308
—— A Pasteur Case, 322
—— Curiosities of Brain and Nerve, 341

Within the Wards:
—— Beef-Tea, and how to make it, 355
—— Diagnosis of Cancer, the, 368
—— About Specialists and Specialism, 404
Woman, a Reasonably Dressed, 265
Women Physicians at Clapham, 402
Women, the New Hospital for, 358
Women, the School of Medicine for, 208
Woolwich and Plumstead Cottage Hospital, 375
Words of Consolation, 272, 288, 304, 320, 336, 352
Working Men Governors at Coventry Hospital, 198
Working Women's Benefit Society, Oxford, 263
Workmen Considering the Establishment of a Hospital, 295
Workmen's Contributions and Hospitals, 33, 79, 109, 230
Worry, Work, and Sunstroke, 377

Young Women's Christian Association and Barmaids, 249

Ziegler, Ernest, Textbook of Pathological Anatomy, 6, 20

### THE SPECIAL NURSING SUPPLEMENT (MIRROR).

America, Nursing in, xix
—— Midwifery in, xcv
Army Nursing, xix
Ashton-on-Mersey, Scheme for District Nursing at, lxxv
Asylum Nurses, xiii, lxxviii, lxxxiii, xcvii, cii
Atcham Board of Guardians and Imbecile Nurses, li, liv
Australia, Notes from, ii, xxiv, xl, lii, lxxx

Bandaging and Surgical Dressing, Mr. Pye on, lxx
Bellevue Hospital, Training School at, lxxxvii
Birmingham General Hospital, Chairman's Speech to Nurses of, lxxxvii, lxxxix
Birmingham Workhouse Infirmary, Troubles at the, xliii
Brighton District Work, lxiii
Bristol District Nurses, ix
*British Medical Journal* on Registration, xliii
British Nurses' Association, xv, xxv, xxviii, xxxiii, xl, li, lix, lx, lxiv
Brutality to Nurses, lxxi
Byron, Harriet Brownlow, lxvii

Carmarthen Infirmary, xxxix
Case Taking, c
Census of Metropolitan Hospital Workers, xxxix
Charing Cross Hospital, Changes at, lxxii
Charity and Religion, xci
Cheerfulness, lxxvi
Children, Sick, xiv
Children's Hospital, Whit Monday in a, xliv
Church Army Nurses, lv
Clapham Maternity Charity, xix
Classes, Maternity and Dispensary, ix
Clean Nails, lxxxii, lxxxvi
Clothing of New-born Children, xiii
Club, a Nurse's, xxxviii, xlv
Combination of Private Nurses, iv, v, xiv
Convalescent Home for Hip Cases, liv
Cooking for Invalids, lv

Deaconesses' Institution, xxxi
—— at Hague, lxiii
—— at Utrecht, lxxix
—— Salaries, lxvii, lxxvii
Deformities in Infancy, xxv
Dentists, Lady, lxxix
Diary, a Nurse's, xxix
Diphtheria, lxxix
Disinfectant, the Best, xxii
District Nursing, xxviii, lxiii, lxvii
Dobree, Miss, a Manual by, lxxxii
Doll Competition, ci
Dress, Women and, v
Dressings, Some New Wound, xli
Dublin Nursing Institution, xlvii

Eastbourne Hospital for Children, lxvii
East London District Nursing Society, Adelaide Ross on the, lxxxiii
Ergot, lxxv
Evelina Hospital, xcv
Examination Questions, lxxxvii, xcviii

First Experience, a, xxxvi, xlviii
Fisher, Miss, and the Philadelphia Hospital, xliii
Food, the Question of, iv, xviii, xxxiv, xlvi

Glebe Hospital for Children at Sydney, Australia, xlvii
Gossip and Nurses, xxxvi
Gravesend Hospital Nursing Institution, xliii
Greenlees, T. D., on the Nursing and Management of the Insane, ii, vi, viii, x, xvi, lxxi
Guardians, Women as, li, liii
Guild at Richmond, a Nursing, lxxxiii

Hague Deaconesses, the, lxiii
Healing Touch, the, xxxii
Hip Cases, Convalescent Home for, liv
Holiday Home, xviii
Homes of Rest for Nurses, xxxiv, xlii

Hospital for Women, the New, li
Hymn for Opening of Hospital, xiii

Imbeciles as Nurses, li, liv
India, Medical Women for, lix
Infection and Uniforms, xxi
Insane, the Care of the, xxxv
Insane, the Nursing and Management of, ii, vi, viii, x, xvi, lxxi, xcix, ci
Institutions, a Warning for, xxxv
Institutions, Nursing, i, xxxi, xxxix, xliii, xlvii, lv, lxvii, lxix, lxxvii
Invalid Children's Aid Association, xiv

Jubilee Nurses' Institution Chaplain, xxxix

*Lancet* on What will Trained Nurses gain by joining the British Nurses' Association, the, xiv, liii
Laughter, the Power of, lxxii
Leicester Children's Hospital, xxxix
Luckes, Miss E. C., on What will Trained Nurses gain by joining the British Nurses' Association, xxxvii

Madeira, Hospital at, xlv
Male Nurses, lxxxvii, xc
Mary Adelaide Nurses, Quarterly Letter to the, li
Marylebone Infirmary, xlvii
Massage, iv, v, xxiii, xxxiv
—— the Weir Mitchell treatment, xci
Maternity Charity, Clapham, xix
Memorial of Nurse Training School Authorities against Registration, lx
Mercier, Dr. Charles, on the Nervous System and the Mind, xxxv
Midwifery in America, xcv
Midwives, and Ophthalmia, xv, xxvi
—— a Warning to, lxiii
—— Institute, the, lxxix
—— Registration of, xiii, xxxi, xxxix
Moral Atmosphere, lix

National Pension Fund for Nurses, v, xx, xlvii, lvii, lix, lxi, lxvi, lxxvii
New Hospital for Women, the, li
New Hospitals, lxvii
New York Nursing, lxvii
—— Training School for Nurses at, xlviii
Nightingale School, the Report of, xlvii
Norfolk and Norwich Hospital, cii
Northampton Nursing Institution, lxix
Nurse, How to Become a, lxxxiv
Nurse-Stewardesses, lxvii
Nurses, To, xc
—— Asylum, xiii, xcvii, cii
Nurse's Bookshelf, the:
a Day's Pleasure for, lxxi
Elementary Bandaging and Surgical Dressing, lxx
Examinations, xlv
Hospital Annual, the, xxx
What will Trained Nurses Gain by Joining the British Nurses' Association, xxxvii
A Manual of Home Nursing, lxxxii
Nurse's Case-Book, the, iv
Nurses' Club, a, xxxviii, xlv
—— Home of Rest, xxxiv, xlii
Nurses and Gossip, xxxvi
—— District, ix, lxvii
—— Imbecile, li, liv
—— Lady, i, v, xiv, xv, xvii
—— Pauper, v
—— Private, iv, v, xv, xxii, lxxviii
—— Combination of Private, iv
—— Off Duty Hours for, xii
—— Recreation, xvii, xix, xxx
—— a Warning to, lxvii
—— the Teaching of, i
Nursing, Army, xix
—— in America, xix
—— at Nice, xxi

Nursing Lectures, ix, xxxv
—— Reports, xxvii
—— Staff, Authority Over, lxiii
—— and Management of the Insane, ii, vi, viii, x, xvi, lxxi, xcix, ci
*Nursing Record* on What will Trained Nurses gain by joining the British Nurses' Association, the, liii
Nursing in the Soudan, iii, vi, x, xvi, xx, xxiv, xxviii

Obedience, xcviii
Operation, a First, l
Ophthalmia and Midwives, xv
Orkney Hospitals, lv
Outings for Nurses, Day, lxx

Pension Fund, the National, v, xx, xlvii, lvii, lix, lxi, lxvi, lxxvii
Pensions, lxxxv
Phthisis and Nurses, lxxix
Physiology for Nurses:
The Skin, lvi, lxi
Baths and their Effects, lxv, lxviii
The Hair and Nails, lxxii
The Skeleton, lxxvi, lxxxi
Spinal Complaints, lxxxv
Joints and Muscles, lxxxviii
Dislocations, xcii
The Mechanism of Movement, xcvi
Poetry, viii, xxxvii, lxxxii, lxxxiv, xc, xciii
Poona Hospital and Mrs. Sheppard, lxxv
Princess of Wales, President of National Pension Fund, lvii, lxvi
Private Nurses, iv, v, xiv, xxii, lxxviii
Probationers, xiv
Probationer's Diary, a, lxxiii, c
Puerperal Fever, xcix
Pye's "Elementary Bandaging and Surgical Dressing," Rev. W., lxx

Recollections of a Nurse, the *Daily News* on the, lxxi
Red Cross in Holland, the, lxvii
Registration of Midwives, xiii, xxxi, xxxix
Registration for Nurses, xl, xliii, xlvii, xlix, li, lvii, lix, lx, lxvi, lxvii, lxix, lxxi lxxiv
Rest for Newcastle Nurses, xix
Richmond, a Nursing Guild at, lxxxiii
Roberts' Nurses' Home and Hospital, Lady, xxxii
Rome, Nurses at, lxxxvii
Royalty and Nursing, xxvii

Salisbury Infirmary, lv
Salvation Army Nurses, lxxv
Sheppard, Mrs., at Poona, lxxv
Sick Nurses at Addenbrooke's, lxiii
Sisters' Uniform, lxxv
Society for Promoting the Return of Ladies as Poor-Law Guardians, the, liii
Soudan, Nursing in the, iii, vi, x, xvi, xx, xxiv, xxviii
Sunderland Sisters, the, lxxvii
Sydney, the Nurses' Home at, lxxvii

Teaching of Nurses, i
Training at Norwich, xv
Training Schools (Nurses) and Registration, lx

Uniforms and Infection, xxi

Vaccination and its Opponents, xi, xviii
Vancouver, St. Luke's Home at, lxxv
Voyages in Vacation, xcvi

Whately's Work, Miss, lxxxvii
Women as Guardians, li, liii
Women, the New Hospital for, li
Women's Parade, a, lxxxvii
Workhouse Infirmary Nursing Association, xvii, xxxvii
Work To Do, vii

Zenana Medical College, xxxvii

### THE STUDENTS' COLUMN.

### "A TEXTBOOK OF PATHOLOGICAL ANATOMY AND PATHOGENESIS."*

The present part of this very valuable and elaborate work comprises the pathological anatomy of the urinary organs, of the central nervous system, and of the peripheral nervous system. The number of subjects treated on is therefore immense, and many of them are illustrated by first-class woodcuts. As evidencing both the number of subjects treated, and the number of diseases to which every part of the human frame is liable, we refer, almost at random, to the chapter on "Maladies of the Nasal Cavities." First, there are congenital malformations, when the nose may be wanting, or only represented by a snout; there are also minor anomalies to the number of about ten. But when a person is fortunate enough to be born with a really good Grecian nose, it becomes in after life quite as subject to disease as the true pug or any other type of the facial appendage. Bleeding from the nose, or *epistaxis*, is very common, and may be due to a great number of causes. Acute nasal catarrh, or *coryza*, or, popularly, "cold in the head," is a complaint familiar to all, and may arise from cold and chill, or from micro-organisms. Chronic nasal catarrh, fortunately, is not so familiar to all, as it occurs chiefly in persons who are scrofulous, phthisical, or syphilitic. The result of this chronic catarrh is very often *ozena*; and the result of this, again, especially in tropical countries, is worms or maggots in the nose. The discharge attracts flies, whose eggs are laid in the nose. The development of larvæ is followed by the destruction of the internal nose, and the conversion of the cavities of the mouth and nose into one chasm. Croupous and diptheritic inflammation are other maladies to which the nose is liable. Syphilitic, tuberculous, and leprous ulcerations are also common, leading to necrosis of bone. Glanders is another disease of the nose. Several kinds of growths, tumours, and polypi form in the organ. *Carcinoma* of the nose is not unfrequently met with; also *rhinoliths* or calcareous concretions, often formed round some foreign body which may have been introduced. There are also vegetable parasites which flourish by preference in the nostrils, the *saccharomyces albicans* being the most common.

The morbid anatomy of these and other maladies is given with great precision, and as showing how thoroughly this is done we quote "'chronic parenchymatous nephritis.' The inflammations of the kidney comprehended under this term are all characterised by persistent exudation from the blood-vessels into the renal tissue, accompanied by alteration of the epithelial structures. The exudation takes place partly from the glomeruli, and partly from the intertubular capillaries and venules. The intertubular exudation saturates the renal tissues with inflammatory lymph, varying in quantity at different stages of the process and in different cases. This inflammatory œdema is always accompanied by a more or less extensive cellular infiltration, which is often remarkably dense around the subcortical and intertubular venules, often, also, well marked in the neighbourhood of the intertubular capillaries, and here and there in exceptional amount round a few of the glomeruli. The extravasated leucocytes and the liquid exudation may penetrate directly into the tubules, and the leucocytes gathered around the Bowman's capsules may in like manner penetrate them. Intertubular venous hæmorrhages are occasionally observed, and when the tubules are at the same time ruptured, blood may enter these directly. The vessels of certain of the glomeruli prevent the escape of albuminous urine, which even during life may coagulate into a granular or homogeneous mass within the capsules. More commonly

* "A Textbook of Pathological Anatomy and Pathogenesis." By Ernest Ziegler, Professor of Pathological Anatomy in the University of Tübingen. Translated and edited for English students by Donal MacAlister, M.A., M.D., Fellow Royal College of Physicians, University Lecturer on Medicine, and Physician to Addenbrookes Hospital, Cambridge. Part II., sect. ix.-xii. Macmillan and Co., London.

coagulation takes place only within the tubules, giving rise to the familiar hyaline casts or cylinders," etc. All this, and much more, is fully illustrated by a carefully-drawn plate, and the authority for everything advanced is quoted.

In the description of multiple sclerosis of the brain and cord, we have as follows: "This is characterised by the formation of a number of grey condensed patches in the nervous tissue. The patches are—some of them superficial, some deep; in the former case they can be recognised by their grey colour. Sometimes they are rounded in shape, sometimes elongated and irregular. Their diameter varies from one millimetre to 50 or more. On section they look uniformly grey and translucent, occasionally one or two are mottled with white, and softer than the others. They are usually sharply defined against the sound tissue, though now and then a patch is surrounded by an ill-defined zone of a greyish white or mottled appearance. In general they are firm and dry, but cases occur in which they are softer than the healthy tissue, and contain a quantity of liquid that escapes on section. The dense patches consist of a close network of delicate sharply-contoured fibres, beset with a number of *nuclei*. Within the larger and firmer patches no nerve fibres can be seen; in the smaller and more recent, or round the border of the larger ones, a few still persist. They are usually normal in appearance, though sometimes they show signs of degeneration. Fat granule cells are in some cases entirely absent, though in general a few can be seen."

*(To be continued.)*

### How to Give Medicine to Children.

### IMPORTANT NOTICE.

The First Chapter of

### A NEW SERIAL STORY

entitled

### Her Heart's Mistake,

By E. D. CUMMING,

*Author of "An Ordeal by Silence," etc.,*

Is commenced in our present issue, being No. 1 of Volume VI.

### A ROYAL NUMBER

Of The Hospital will be published on April 27th, 1889. It will contain an account of the Public Life and Work of their Royal Highnesses

THE PRINCE AND PRINCESS OF WALES.

*** The monthly part for May will contain Special Portraits of their Royal Highnesses. See notice below as to separate copies.

Intending Subscribers should send their names without delay to the Publisher, The Hospital, 139-140, Salisbury Court, Fleet Street, E.C.

OUR NEW OFFICES,

139 and 140, SALISBURY COURT,

FLEET STREET, E.C.

☞ Our readers are asked to note that with the new volume (which begins this week) portraits of leaders in the medical, philanthropic and nursing world will be issued with the monthly parts, the first of which will be ready on April 30th. For the convenience of weekly subscribers, *these portraits will be issued separately, at 3d. each, but must be ordered at an early date,* to secure prompt delivery on date of publication. In consequence of the growing requirements of The Hospital, and of the inconvenience to the trade arising from the distance of our old offices from the publishing centre, it has been found necessary to remove to the above address.

### Scraps and Gleanings.

The lunatics in Paris number over 8,000.

There is an epidemic of measles at Aberdeen.

A Clinical Society has been formed at Ipswich.

Lisbon is suffering from an epidemic of typhoid.

A Medical Congress is being organised at Havana.

There are about 150 American medical students at Vienna.

A cabman's club and reading-room exists in Westbourne-grove.

There are two cases of smallpox on the hospital ships at present.

The workmen of Jarrow subscribe £20 a week to their accident hospital.

The number of candidates for membership of the Royal Society is 71 this year.

The mean mortality in the urban population of Belgium last year was 21·2 per 1,000.

The fresco of Spring at Guy's Hospital is the work of Mr. Herbert James Draper.

Dr. James Little has been elected consulting-physician to Steevens Hospital, Dublin.

The Municipality of Berlin intends to create a new establishment for 700 epileptic patient.

A monthly calendar of services, etc., is now issued by the Chaplain of the London Hospital.

Mr. G. S. Pope has been appointed assistant medical officer to the Friends' Retreat, York.

Sir Edward Sieveking presided at the late meeting of the St. John Ambulance Association.

The Vicar of St. Agnes, Kennington Park, appeals for funds for the completion of his church.

The Dodworth Hospital Sunday Committee have handed a cheque for £36 to the Bechett Hospital.

The number of paupers in our workhouses is beginning to decrease as the milder weather comes on.

Professor Bellroth, of Vienna, lately successfully removed a tumour which weighed 19 kilogrammes.

Dr. Townsend has been appointed Professor of the Practice of Medicine at Queen's College, Cork.

Over one-third of the annual total of deaths in Liverpool are caused by consumption and chest diseases.

The Hon. Miss Sugden, sister of Lord St. Leonards, has charge of the Medical Mission at Hankow, China.

Mdlle. Richaud has been fined 30 francs for dispensing atropine instead of antipyrin; the patient died.

The Japanese Empress proposes to visit America attended, amongst others, by her dentist and ten doctors.

Twelve thousand out-patients to Leicester Infirmary rather looks as though that charity were being abused.

The correspondents of the *Richmond Herald* are still fighting the question of the Mortlake Fever Hospital.

The death is announced at Cannes of Dr. Charles J. B. Williams, a once famous consulting physician in London.

The Preston Infirmary urges its claim upon the people of Fleetwood, many of whom it has cared for in serious illness.

Miss Emily L. Gregory, Ph.D., has been appointed a fellow of the Biological Department of Pennsylvania University.

Belfast Ophthalmic Hospital treated 1,500 out-patients and 111 in-patients last year. There is a balance in hand of £84.

Sir James Paget presided last week at the annual meeting of the Marylebone and Paddington District Nursing Association.

A statue has been erected at Hobart, Tasmania, to the memory of the Hon. W. L. Crowther, at one time surgeon to Hobart Hospital.

Miss Lucy Cradock, medical officer of female clerks in Liverpool Post Office, has been elected a member of the Liverpool Medical Society.

Mr. Felix Joseph has presented a handsome clock, fitted with electric light apparatus, to the Princess Alice Memorial Hospital, Eastbourne.

Mr. Joseph Bormond, the well-known teetotaller, died last week at the Temperance Hospital from the effects of a fall. He was over 80 years of age.

The Nottingham Samaritan Hospital surgeons performed 73 operations last year, of which 30 were abdominal sections. The death-rate was $1\frac{1}{2}$ per cent.

It is proposed to build a postman's rest at Paddington. The idea is a good one, and we recommend it to those who are both practical and charitable.

Monkstown Hospital, Dublin, is to have an "Observation Ward where doubtful or contagious cases can be kept for a while. The title s a good one.

Dr. Mary Stinson has died in Pennsylvania at the age of 70. Apparently she found professional labours not beyond the physical powers of her sex.

A deputation of the Northampton Hospital Week Fund have been visiting Rushden and other places, and holding meetings with a view to arousing interest in the prosperity of hospitals.

The late Mrs. L. E. V. Harcourt, 3, Minster-court, York, and Bridlington Quay, leaves by her will on the death of her sister Mrs. Charlotte Egerton, £10,000 to the Bridlington Convalescent Home.

By the resignation of Mr. C. E. Pronger, and the death of Mr. H. Jackson, there are two vacancies on the medical staff of the North Devon Infirmary. The vacancies will be filled up on April 27th.

A Bill to exempt charities and hospitals from local rates has been read a first time in the House of Commons. It is backed by Sir Julian Goldsmid, Baron de Rothschild, Sir Robert Fowler, Sir Algernon Borthwick, Mr. Octavius V. Morgan, and Mr. Lawson.

### Amusements and Relaxation.

NEW PUZZLE PRIZE SCHEME.]

THIRD QUARTERLY COMPETITION COMMENCED APRIL 4TH 1889, ENDS JUNE 27TH, 1889.

### SPECIAL NOTICE.

A New Original Acrostic Competition was commenced in these columns on Thursday, April 4.

Acrostics to be sent in every fortnight, commencing Thursday, April 4th, and ending June 27th.

Two prizes will be given—First Prize of Half-a-Guinea, and Second Prize of Five Shillings.

Acrostics will be credited according to merit, six marks being the highest score given for any one Acrostic.

In addition to the above prizes, one of *Half a Guinea* will be given for Solution of Acrostics set.

One mark only will be given for the correct solution of all lights of each Acrostic.

The first Acrostic for solution will be given on Saturday next, April 13th.

### Word Competition.

At the request of many of our readers we have decided to start another weekly Word Dissection, commencing April 6th, and ending June 29th.

Three Prizes of 15s., 10s., 5s., will be given for the largest number of words derived from the word set for dissection.

Proper names, abbreviations, foreign words, words of less than four letters, and repetitions are barred; plurals, and past and present participles of verbs, are allowed. Nuttall's *Standard* dictionary only to be used.

N.B.—All words sent in must be arranged *alphabetically*, with correct total affixed.

The word for dissection for this, the first week of the quarter, being

"BOULANGER.'

### Marks for Original Puzzles.

| Names | No. of Puzzles. sent in Mar. 28. | No. of Marks. Mar. 28. | Totals. | Names. | No. of Puzzles sent in Mar. 28. | No. of Marks. Mar. 28. | Totals. |
|---|---|---|---|---|---|---|---|
| Cotonopolis | 1 | 3 | 48 | Prior ...... | 3 | 2 | 28 |
| Tinie ...... | 3 | 5 | 59 | Lightowlers | 3 | 3 | 33 |
| Jenny Wren | — | — | 7 | Twentieth.. | — | — | 3 |
| Patience .. | 3 | 7 | 70 | Crocodile .. | 1 | 3 | 17 |
| Padre...... | — | — | 3 | Nabob .... | 1 | 3 | 20 |

### Answers.

No. 25. Double Acrostic.

P andemoniu M
O meg A
L egio N
L apis Lazu I
O asi S
C el T
K e Y

No. 26. Original Anagrams.

1. The Parnell Commission.
2. The Famine in China.

### Marks for Solution of Puzzles.

Total Marks.—Flora (1), 13; Mater (1), 10; Scrooge (1), 13; Patience 4; Lady Clara (1), 14; Ruffles, 9; Lauriston (1), 4; Reldas, 9; Jenny Wren, 5; Condorcet, 2; Tinie, 2; Daffodil, 1.

[*The Figures in parentheses are Marks for the week ending March 28th.*

### Notice to Correspondents.

RESULTS OF SECOND QUARTERLY COMPETITION WILL BE PUBLISHED NEXT WEEK.

### Conditions.

I. Every paper sent in must be clearly written, and one side only must be used. All competitors must mark at the head of their papers the number of their competition.

II. Each paper must be signed by the author with his or her real name and address. A *nom de plume* may be added if the writer does not desire to be referred to by us by his real name. In the case of all prizewinners, however, the real name and address will be published.

III. All communications relating to prize competitions should be addressed to "The Prize Editor, The Hospital, 139 and 140, Salisbury-court, London, E.C." Competitors are requested not to include in letters, etc., to the Prize Editor communications on matters of other business. All letters must be fully prepaid.

IV. The Prize Editor of The Hospital is the sole judge of the competitions, and his decision is in all cases final and without appeal.

V. The Prize Editor reserves the right to publish any paper sent in, whether it receives a prize or not. He does not hold himself responsible for any MSS. sent, nor can he undertake to return rejected competitions.

